# Supplementary material for: Hybrid Models and Biological Model Reduction with PyDSTool
Source: PLoS Comput Biol. 2012 Aug 9;8(8):e1002628. doi: 10.1371/journal.pcbi.1002628 (PMC3415397; doi:10.1371/journal.pcbi.1002628)
Supplement: Text S4 — Complete source code for the PyDSTool package (version 0.88.120504). Includes API documentation and help files linking to web pages. This file is identical to the current public release on Sourceforge.net. (ZIP) [file pcbi.1002628.s004.zip › PyDSTool/html/PyDSTool.PyCont.Plotting-pysrc.html]

xml version="1.0" encoding="ascii"?


PyDSTool.PyCont.Plotting


| Home | Trees | Indices | Help | PyDSTool |
| --- | --- | --- | --- | --- |

Package PyDSTool ::
Package PyCont ::
Module Plotting

[hide private]

[frames] | no frames]

# Source Code for Module PyDSTool.PyCont.Plotting

```
  1  """ Plotting class and function 
  2   
  3      Drew LaMar, May 2006 
  4  """ 
  5   
  6  # ---------------------------------------------------------------------------- 
  7  # Version History: 
  8  #   February 2007 
  9  #       Works with SciPy 0.5.2 
 10  # 
 11  #   September 2006 
 12  #       Added get method 
 13  #       BUG FIX: refresh() now refreshes figures not axes (didn't work with axes) 
 14  #           This is a Subplot issue. 
 15  #       BUG FIX: set title of axes was calling pylab instead of directly 
 16  #       Added KeyEvent class used by 'highlight' method in plot_cycles 
 17  # 
 18  #   June 2006 
 19  #       Created pargs class and initializeDisplay method 
 20  # 
 21  # ---------------------------------------------------------------------------- 
 22   
 23  from PyDSTool.common import args 
 24  from PyDSTool.matplotlib_import import * 
 25   
 26  # THESE ARE REPEATS FROM CONTINUATION!  MAKE SURE AND UPDATE!!! 
 27  all_point_types = ['P', 'RG', 'LP', 'BP', 'H', 'BT', 'ZH', 'CP', 'GH', 'DH', 'LPC', 'PD', 
 28                     'NS', 'MX', 'UZ', 'B'] 
 29  all_curve_types = ['EP', 'LP', 'H', 'FP', 'LC'] 
 30   
 31  ##### 
 32  _classes = ['pargs', 'KeyEvent'] 
 33   
 34  _functions = ['initializeDisplay'] 
 35   
 36  __all__ = _classes + _functions 
 37  ##### 
 38   


39 -class pargs(args):


40 -    def __getitem__(self, key):


41          if len(key.split(':')) > 1: 
 42              (curve, pt) = key.split(':') 
 43              return args.__getitem__(args.__getitem__(self, curve), pt) 
 44          else: 
 45              return args.__getitem__(self, key)

 46   


47 -    def __repr__(self, ct=0, res=None):


48          if res is None: 
 49              res = [] 
 50   
 51          # This is so I can make things alphabetical 
 52          all_pargs = [] 
 53          all_args = [] 
 54          deleted = self.has_key('deleted') and self.deleted 
 55          if not deleted: 
 56              for k, v in self.iteritems(): 
 57                  if k in ['point', 'curve', 'cycle']: 
 58                      if v[0].get_label() != '_nolegend_': 
 59                          all_args.append(('Legend', v[0].get_label())) 
 60                  elif k == 'text': 
 61                      if v is not None: 
 62                          all_args.append(('Label', v.get_text().lstrip(' '))) 
 63                  elif k == 'type': 
 64                      all_args.append(('Type', self['type'])) 
 65                  elif isinstance(v, pargs): 
 66                      all_pargs.append((k, v)) 
 67   
 68          if len(all_args) == 0 and len(all_pargs) == 0: 
 69              if self.has_key('deleted'): 
 70                  res.append(ct*4*' ' + 'Deleted\n') 
 71              else: 
 72                  res.append(ct*4*' ' + 'Empty\n') 
 73          else: 
 74              all_args.sort() 
 75              if len(all_args) > 0: 
 76                  for n in all_args: 
 77                      res.append(ct*4*' ' + n[0] + ': ' + n[1] + '\n') 
 78   
 79              all_pargs.sort() 
 80              if len(all_pargs) > 0: 
 81                  ct += 1 
 82                  for n in all_pargs: 
 83                      res.append((ct-1)*4*' ' + n[0] + ':' + '\n') 
 84                      n[1].__repr__(ct=ct, res=res) 
 85   
 86          if ct <= 1: 
 87              return reduce(lambda x, y: x+y, res)

 88   
 89      __str__ = __repr__ 
 90   


91 -    def fromLabel(self, label, key=None):


92          point = None 
 93          if self.has_key('text') and self.text.get_text().lstrip(' ') == label or \ 
 94             self.has_key('point') and self.point[0].get_label() == label or \ 
 95             self.has_key('curve') and self.curve[0].get_label() == label: 
 96              return (key, self) 
 97          for k, v in self.iteritems(): 
 98              if isinstance(v, pargs): 
 99                  point = v.fromLabel(label, key=k) 
100                  if point is not None: 
101                      break 
102          return point

103   


104 -    def get(self, objtype, bylabel=None, byname=None, bytype=None, bylegend=None, obj=None, ct=0):


105          if objtype not in ['point', 'text', 'cycle', 'curve']: 
106              raise 'Object type must be point, text, cycle, or curve' 
107   
108          ct += 1 
109          if isinstance(bylabel, str): 
110              bylabel = [bylabel] 
111          if isinstance(byname, str): 
112              byname = [byname] 
113          if isinstance(bytype, str): 
114              bytype = [bytype] 
115          if isinstance(bylegend, str): 
116              bylegend = [bylegend] 
117   
118          if obj is None: 
119              obj = [] 
120          if bylabel is None and byname is None and bytype is None and bylegend is None: 
121              if objtype in ['point', 'text', 'cycle']: 
122                  bytype = all_point_types 
123              elif objtype  == 'curve': 
124                  bytype = all_curve_types 
125          for k, v in self.iteritems(): 
126              if isinstance(v, pargs): 
127                  if v.has_key(objtype): 
128                      if bylabel is not None and v.has_key('text') and v.text.get_text().lstrip(' ') in bylabel or \ 
129                         byname is not None and k in byname or \ 
130                         bytype is not None and k.strip('0123456789') in bytype or \ 
131                         bylegend is not None and ((v.has_key('curve') and v.curve[0].get_label() in bylegend) or (v.has_key('cycle') and v.cycle[0].get_label() in bylegend)): 
132                             obj.append((k,v[objtype])) 
133                  v.get(objtype, bylabel=bylabel, byname=byname, bytype=bytype, bylegend=bylegend, obj=obj, ct=ct) 
134   
135          if ct == 1: 
136              return obj

137   


138 -    def toggleLabel(self, visible='on', refresh=True):


139          if self.has_key('text'): 
140              self.text.set_visible(visible == 'on') 
141   
142          if refresh: 
143              self.refresh()

144   


145 -    def toggleLabels(self, visible='on', bylabel=None, byname=None, bytype=None, ct=0):


146          ct += 1 
147          if isinstance(bylabel, str): 
148              bylabel = [bylabel] 
149          if isinstance(byname, str): 
150              byname = [byname] 
151          if isinstance(bytype, str): 
152              bytype = [bytype] 
153   
154          if bylabel is None and byname is None and bytype is None: 
155              bytype = all_point_types    # Turn off all points 
156          for k, v in self.iteritems(): 
157              if bytype is not None and k.strip('0123456789') in bytype or \ 
158                 byname is not None and k in byname or \ 
159                 bylabel is not None and isinstance(v, pargs) and v.has_key('text') and v.text.get_text().lstrip(' ') in bylabel: 
160                  v.toggleLabel(visible=visible, refresh=False) 
161              elif isinstance(v, pargs): 
162                  v.toggleLabels(visible=visible, bylabel=bylabel, byname=byname, bytype=bytype, ct=ct) 
163   
164          if ct == 1: 
165              self.refresh()

166   


167 -    def togglePoint(self, visible='on', refresh=True):


168          if self.has_key('point'): 
169              self.point[0].set_visible(visible == 'on') 
170   
171          if refresh: 
172              self.refresh()

173   


174 -    def togglePoints(self, visible='on', bylabel=None, byname=None, bytype=None, ct=0):


175          ct += 1 
176          if isinstance(bylabel, str): 
177              bylabel = [bylabel] 
178          if isinstance(byname, str): 
179              byname = [byname] 
180          if isinstance(bytype, str): 
181              bytype = [bytype] 
182   
183          if bylabel is None and byname is None and bytype is None: 
184              bytype = all_point_types    # Turn off all points 
185          for k, v in self.iteritems(): 
186              if bytype is not None and k.strip('0123456789') in bytype or \ 
187                 byname is not None and k in byname or \ 
188                 bylabel is not None and isinstance(v, pargs) and v.has_key('text') and v.text.get_text().lstrip(' ') in bylabel: 
189                  v.togglePoint(visible=visible, refresh=False) 
190              elif isinstance(v, pargs): 
191                  v.togglePoints(visible=visible, bylabel=bylabel, byname=byname, bytype=bytype, ct=ct) 
192   
193          if ct == 1: 
194              self.refresh()

195   


196 -    def toggleCurve(self, visible='on', refresh=True):


197          if self.has_key('curve'): 
198              for k, v in self.iteritems(): 
199                  if isinstance(v, pargs): 
200                      v.toggleLabel(visible=visible, refresh=False) 
201                      v.togglePoint(visible=visible, refresh=False) 
202              for line in self.curve: 
203                  line.set_visible(visible == 'on') 
204   
205          if refresh: 
206              self.refresh()

207   


208 -    def toggleCurves(self, visible='on', bylegend=None, byname=None, bytype=None, ct=0):


209          ct += 1 
210          if isinstance(bylegend, str): 
211              bylegend = [bylegend] 
212          if isinstance(byname, str): 
213              byname = [byname] 
214          if isinstance(bytype, str): 
215              bytype = [bytype] 
216   
217          if bylegend is None and byname is None and bytype is None: 
218              bytype = all_curve_types 
219          for k, v in self.iteritems(): 
220              if bytype is not None and isinstance(v, pargs) and v.has_key('type') and v.type in bytype or \ 
221                 byname is not None and k in byname or \ 
222                 bylegend is not None and isinstance(v, pargs) and v.has_key('curve') and v.curve[0].get_label() in bylegend: 
223                  v.toggleCurve(visible=visible, refresh=False) 
224              elif isinstance(v, pargs): 
225                  v.toggleCurves(visible=visible, bylegend=bylegend, byname=byname, bytype=bytype, ct=ct) 
226   
227          if ct == 1: 
228              self.refresh()

229   


230 -    def toggleCycle(self, visible='on', refresh=True):


231          if self.has_key('cycle'): 
232              for line in self.cycle: 
233                  line.set_visible(visible == 'on') 
234   
235          if refresh: 
236              self.refresh()

237   


238 -    def toggleCycles(self, visible='on', bylegend=None, byname=None, bytype=None, ct=0):


239          ct += 1 
240          if isinstance(bylegend, str): 
241              bylegend = [bylegend] 
242          if isinstance(byname, str): 
243              byname = [byname] 
244          if isinstance(bytype, str): 
245              bytype = [bytype] 
246   
247          if bylegend is None and byname is None and bytype is None: 
248              bytype = all_point_types 
249          for k, v in self.iteritems(): 
250              if bytype is not None and k.strip('0123456789') in bytype or \ 
251                 byname is not None and k in byname or \ 
252                 bylegend is not None and isinstance(v, pargs) and v.has_key('cycle') and v.cycle[0].get_label() in bylegend: 
253                  v.toggleCycle(visible=visible, refresh=False) 
254              elif isinstance(v, pargs): 
255                  v.toggleCycles(visible=visible, bylegend=bylegend, byname=byname, bytype=bytype, ct=ct) 
256   
257          if ct == 1: 
258              self.refresh()

259   


260 -    def toggleAll(self, visible='on', bylabel=None, byname=None, bytype=None, ct=0):


261          ct += 1 
262          if isinstance(bylabel, str): 
263              bylabel = [bylabel] 
264          if isinstance(byname, str): 
265              byname = [byname] 
266          if isinstance(bytype, str): 
267              bytype = [bytype] 
268   
269          if bylabel is None and byname is None and bytype is None: 
270              bytype = all_point_types 
271          for k, v in self.iteritems(): 
272              if bytype is not None and k.strip('0123456789') in bytype or \ 
273                 byname is not None and k in byname or \ 
274                 bylabel is not None and isinstance(v, pargs) and ((v.has_key('text') and v.text.get_text().lstrip(' ') in bylabel) or (v.has_key('curve') and v.curve[0].get_label() in bylabel) or (v.has_key('cycle') and v.cycle[0].get_label() in bylabel)): 
275                  v.toggleLabel(visible=visible, refresh=False) 
276                  v.togglePoint(visible=visible, refresh=False) 
277                  v.toggleCycle(visible=visible, refresh=False) 
278              elif isinstance(v, pargs): 
279                  v.toggleAll(visible=visible, bylabel=bylabel, byname=byname, bytype=bytype, ct=ct) 
280   
281          if ct == 1: 
282              self.refresh()

283   


284 -    def setLabel(self, label, refresh=True):


285          if self.has_key('text'): 
286              self.text.set_text('  '+label) 
287   
288          if refresh: 
289              self.refresh()

290   


291 -    def setLabels(self, label, bylabel=None, byname=None, bytype=None, ct=0):


292          ct += 1 
293          if isinstance(bylabel, str): 
294              bylabel = [bylabel] 
295          if isinstance(byname, str): 
296              byname = [byname] 
297          if isinstance(bytype, str): 
298              bytype = [bytype] 
299   
300          if bylabel is None and byname is None and bytype is None: 
301              bytype = all_point_types    # Turn off all points 
302          for k, v in self.iteritems(): 
303              if bytype is not None and k.strip('0123456789') in bytype or \ 
304                 byname is not None and k in byname or \ 
305                 bylabel is not None and isinstance(v, pargs) and v.has_key('text') and v.text.get_text().lstrip(' ') in bylabel: 
306                  v.setLabel(label, refresh=False) 
307              elif isinstance(v, pargs): 
308                  v.setLabels(label, bylabel=bylabel, byname=byname, bytype=bytype, ct=ct) 
309   
310          if ct == 1: 
311              self.refresh()

312   


313 -    def setLegend(self, legend, refresh=True):


314          if self.has_key('curve'): 
315              for piece in self.curve: 
316                  piece.set_label(legend) 
317          elif self.has_key('cycle'): 
318              for piece in self.cycle: 
319                  piece.set_label(legend) 
320   
321          if refresh: 
322              self.refresh()

323   


324 -    def setLegends(self, legend, bylegend=None, byname=None, bytype=None, ct=0):


325          ct += 1 
326          if isinstance(bylegend, str): 
327              bylabel = [bylegend] 
328          if isinstance(byname, str): 
329              byname = [byname] 
330          if isinstance(bytype, str): 
331              bytype = [bytype] 
332   
333          if bylegend is None and byname is None and bytype is None: 
334              if v.has_key('curve'): 
335                  bytype = all_curve_types 
336              else: 
337                  bytype = all_point_types    # Turn off all points 
338          for k, v in self.iteritems(): 
339              if bytype is not None and isinstance(v, pargs) and ((v.has_key('curve') and v.type in bytype) or (v.has_key('cycle') and k.strip('0123456789') in bytype)) or \ 
340                 byname is not None and k in byname or \ 
341                 bylegend is not None and isinstance(v, pargs) and ((v.has_key('curve') and v.curve[0].get_label() in bylegend) or (v.has_key('cycle') and v.cycle[0].get_label() in bylegend)): 
342                  v.setLegend(legend, refresh=False) 
343              elif isinstance(v, pargs): 
344                  v.setLegends(legend, bylegend=bylegend, byname=byname, bytype=bytype, ct=ct) 
345   
346          if ct == 1: 
347              self.refresh()

348   


349 -    def refresh(self):


350          fig = None  # Used to save current figure (reset when done) 
351   
352          if not self.has_key('deleted'): 
353              if 'fig' in self.keys(): 
354                  pylab.figure(self.fig.number) 
355                  pylab.draw() 
356              elif 'axes' in self.keys(): 
357                  pylab.figure(self.axes.figure.number) 
358                  #Would like to say: pylab.axes(self.axes)  Doesn't work, though. 
359                  pylab.draw() 
360              elif 'curve' in self.keys(): 
361                  pylab.figure(self.curve[0].figure.number) 
362                  pylab.draw() 
363              elif 'point' in self.keys(): 
364                  pylab.figure(self.point[0].figure.number) 
365                  pylab.draw() 
366              elif 'cycle' in self.keys(): 
367                  pylab.figure(self.cycle[0].figure.number) 
368                  pylab.draw() 
369              else:   # Only here in plot structure 
370                  fig = pylab.gcf() 
371                  for k, v in self.iteritems(): 
372                      if isinstance(v, pargs): 
373                          v.refresh() 
374   
375          if fig is not None: # Reset to current figure 
376              pylab.figure(fig.number)

377   


378 -    def clean(self):


379          """Cleans plotting structure (e.g. deleted)  Also gets rid of nonexisting figures 
380          if they've been deleted by an outside source.  This method is a little clumsy since 
381          it has to spawn a dummy plot if a plot has been deleted, but who cares, it works.""" 
382          deleted = [] 
383          for k, v in self.iteritems(): 
384              recursive_clean = True 
385              if isinstance(v, pargs): 
386                  if v.has_key('deleted'): 
387                      deleted.append(k) 
388                      recursive_clean = False 
389                  elif v.has_key('fig'): 
390                      fig_check = pylab.figure(v.fig.number) 
391                      if fig_check != v.fig: 
392                          pylab.close(fig_check) 
393                          deleted.append(k) 
394                          recursive_clean = False 
395                  elif v.has_key('axes'): 
396                      try: 
397                          fig_axes = pylab.axes(v.axes) 
398                      except: 
399                          fig_axes = None 
400   
401                      if fig_axes is None: 
402                          deleted.append(k) 
403                          recursive_clean = False 
404                      elif fig_axes not in v.axes.figure.axes: 
405                          print 'Warning:  Axes were deleted without using pylab.delaxes().' 
406                          v.axes.figure.axes.append(fig_axes) 
407                          pylab.draw() 
408                  elif v.has_key('curve'): 
409                      for piece in v.curve: 
410                          if piece not in piece.axes.lines: 
411                              deleted.append(k) 
412                              v.delete()    # Remove remaining pieces of curve in pylab 
413                              recursive_clean = False 
414                              break 
415                  elif v.has_key('cycle'): 
416                      for piece in v.cycle: 
417                          if piece not in piece.axes.lines: 
418                              deleted.append(k) 
419                              v.delete() 
420                              recursive_clean = False 
421                              break 
422                  elif v.has_key('point'): 
423                      if v.point[0] not in v.point[0].axes.lines or v.text not in v.text.axes.texts: 
424                          deleted.append(k) 
425                          if v.text in v.text.axes.texts: 
426                              v.text.axes.texts.remove(v.text) 
427                          if v.point[0] in v.point[0].axes.lines: 
428                              v.point[0].axes.lines.remove(v.point[0]) 
429                          recursive_clean = False 
430   
431                  if recursive_clean: 
432                      v.clean() 
433   
434          for k in deleted: 
435              self.pop(k)

436   


437 -    def clear(self, refresh=True):


438          if not self.has_key('deleted'): 
439              if self.has_key('fig'): 
440                  pylab.figure(self.fig.number) 
441                  pylab.clf() 
442                  remove = [k for k in self.keys() if k != 'fig'] 
443              elif self.has_key('axes'): 
444                  title = self.axes.title.get_text() 
445                  self.axes.clear() 
446                  pylab.axes(self.axes) 
447                  pylab.title(title) 
448                  remove = [k for k in self.keys() if k != 'axes'] 
449                  if refresh: 
450                      self.refresh() 
451              elif self.has_key('curve'): 
452                  remove = [k for k in self.keys() if k != 'curve'] 
453                  for k in remove: 
454                      if isinstance(self[k], pargs): 
455                          self[k].clear(refresh=False) 
456                  if refresh: 
457                      self.refresh() 
458              elif self.has_key('point'): 
459                  if self.point[0] in self.point[0].axes.lines: 
460                      self.point[0].axes.lines.remove(self.point[0]) 
461                  if self.text in self.point[0].axes.texts: 
462                      self.point[0].axes.texts.remove(self.text) 
463                  remove = [k for k in self.keys() if k != 'point'] 
464                  if refresh: 
465                      self.refresh() 
466                  self.deleted = True 
467              else: 
468                  remove = [] 
469   
470              if remove != []: 
471                  for k in remove: 
472                      self.pop(k) 
473          else: 
474              print 'Object is deleted.'

475   


476 -    def clearall(self):


477          for v in self.itervalues(): 
478              if isinstance(v, pargs): 
479                  v.clear(refresh=False) 
480          self.refresh()

481   


482 -    def delete(self, refresh=True):


483          if not self.has_key('deleted'): 
484              if self.has_key('fig'): 
485                  self.clear(refresh=False) 
486                  pylab.close() 
487                  self.deleted = True 
488              elif self.has_key('axes'): 
489                  self.clear(refresh=False) 
490                  pylab.delaxes() 
491                  self.deleted = True 
492              elif self.has_key('curve'): 
493                  self.clear(refresh=False) 
494                  for curve in self.curve: 
495                      if curve in self.curve[0].axes.lines: 
496                          self.curve[0].axes.lines.remove(curve) 
497                  if refresh: 
498                      self.refresh() 
499                  self.deleted = True 
500              elif self.has_key('cycle'): 
501                  self.clear(refresh=False) 
502                  for cycle in self.cycle: 
503                      if cycle in self.cycle[0].axes.lines: 
504                          self.cycle[0].axes.lines.remove(cycle) 
505                  if refresh: 
506                      self.refresh() 
507                  self.deleted = True 
508              elif self.has_key('point'): 
509                  self.clear(refresh=refresh) 
510              else: 
511                  for v in self.itervalues(): 
512                      if isinstance(v, pargs): 
513                          v.delete(refresh=refresh) 
514          else: 
515              print 'Object is already deleted.'

516   


517 -    def deleteall(self):


518          for v in self.itervalues(): 
519              if isinstance(v, pargs): 
520                  v.delete(refresh=False) 
521          self.refresh()

522   


523 -class KeyEvent(object):


524      """Used in 'highlight' method of plot_cycles.""" 


525 -    def __init__(self, paxes):


526          self.curr = 0 
527          self.axes = paxes.axes 
528   
529          # Get list of cycles from pargs class axes 
530          cycles = [thing.cycle[0] for k, thing in paxes.iteritems() if isinstance(thing, pargs) and thing.has_key('cycle')] 
531          # Put in order as in axes.lines (this should be same as order along curve, or user 
532          #   specified in cycles when plot_cycles was called) 
533          self.cycles = [] 
534          for line in self.axes.lines: 
535              if line in cycles: 
536                  self.cycles.append(line) 
537          self.axes.set_title(self.cycles[0].get_label()) 
538   
539          self.bgd_lw = 0.2 
540          self.fgd_lw = 1.5 
541          for line in self.cycles: 
542              line.set(linewidth=self.bgd_lw) 
543          self.cycles[0].set(linewidth=self.fgd_lw) 
544          pylab.draw() 
545          pylab.connect('key_press_event', self.__call__)

546   


547 -    def __call__(self, event):


548          if event.key == 'right': self.change_curr(1) 
549          elif event.key == 'left': self.change_curr(-1) 
550          elif event.key == 'up': self.change_bgd(1) 
551          elif event.key == 'down': self.change_bgd(-1) 
552          elif event.key in ('+', '='): self.change_fgd(1) 
553          elif event.key in ('-', '_'): self.change_fgd(-1)

554   


555 -    def change_bgd(self, up):


556          if up == 1: 
557              if self.bgd_lw+0.1 > self.fgd_lw: 
558                  self.bgd_lw = self.fgd_lw 
559              else: 
560                  self.bgd_lw += 0.1 
561          else: 
562              if self.bgd_lw-0.1 < 0: 
563                  self.bgd_lw = 0 
564              else: 
565                  self.bgd_lw -= 0.1 
566   
567          for ct, line in enumerate(self.cycles): 
568              if ct != self.curr: 
569                  line.set(linewidth=self.bgd_lw) 
570          pylab.draw() 
571   
572          print 'Background linewidth = %f' % self.bgd_lw

573   


574 -    def change_fgd(self, up):


575          if up == 1: 
576              self.fgd_lw += 0.1 
577          else: 
578              if self.fgd_lw-0.1 < self.bgd_lw: 
579                  self.fgd_lw = self.bgd_lw 
580              else: 
581                  self.fgd_lw -= 0.1 
582   
583          self.cycles[self.curr].set(linewidth=self.fgd_lw) 
584          pylab.draw() 
585   
586          print 'Foreground linewidth = %f' % self.fgd_lw

587   


588 -    def change_curr(self, up):


589          self.cycles[self.curr].set(linewidth=self.bgd_lw) 
590          if up == 1: 
591              self.curr = (self.curr+1) % len(self.cycles) 
592          else: 
593              self.curr = (self.curr-1) % len(self.cycles) 
594          self.cycles[self.curr].set(linewidth=self.fgd_lw) 
595          self.axes.set_title(self.cycles[self.curr].get_label()) 
596          pylab.draw()

597   


598 -def initializeDisplay(plot, figure=None, axes=None):


599      """If figure = 'new', then it will create a new figure with name fig# 
600      If figure is an integer, that figure # will be selected 
601      """ 
602   
603      plot.clean()   # Clean up plot structure 
604      plot._cfl = None 
605      plot._cal = None 
606   
607      # Handle figure 
608      if figure is None: 
609          if len(plot) <= 3: 
610              figure = pylab.gcf() 
611          else: 
612              raise ValueError('Please specify a figure.') 
613   
614      cfl = None 
615      if isinstance(figure, pylab.Figure): 
616          for k, v in plot.iteritems(): 
617              if isinstance(v, pargs) and v.fig == figure: 
618                  cfl = k 
619                  break 
620      elif isinstance(figure, str): 
621          if figure == 'new': 
622              figure = pylab.figure() 
623          else: 
624              cfl = figure 
625              if cfl not in plot.keys(): 
626                  plot[cfl] = pargs() 
627                  plot[cfl].fig = pylab.figure() 
628      elif isinstance(figure, int): 
629          fighandle = pylab.figure(figure) 
630          cfl = 'fig' + str(figure) 
631          if cfl not in plot.keys(): 
632              plot[cfl] = pargs() 
633              plot[cfl].fig = fighandle 
634      else: 
635          raise TypeError("Invalid type for figure argument") 
636   
637      if cfl is None: 
638          cfl = 'fig1' 
639          ct = 1 
640          while cfl in plot.keys(): 
641              ct += 1 
642              cfl = 'fig'+repr(ct) 
643          plot[cfl] = pargs() 
644          plot[cfl].fig = figure 
645   
646      pylab.figure(plot[cfl].fig.number) 
647   
648      # Handle axes 
649      if not axes: 
650          if len(plot[cfl]) <= 2: 
651              axes = pylab.gca() 
652          else: 
653              raise 'Please specify axes.' 
654      elif isinstance(axes, tuple): 
655          if len(axes) == 3: 
656              axes = pylab.subplot(axes[0],axes[1],axes[2]) 
657          else: 
658              raise 'Tuple must be of length 3' 
659   
660      cal = None 
661      if isinstance(axes, pylab.Axes): 
662          for k, v in plot[cfl].iteritems(): 
663              if isinstance(v, pargs) and v.axes == axes: 
664                  cal = k 
665                  break 
666   
667          if cal is None: 
668              cal = 'axes1' 
669              ct = 1 
670              while cal in plot[cfl].keys(): 
671                  ct += 1 
672                  cal = 'axes'+repr(ct) 
673              plot[cfl][cal] = pargs() 
674              plot[cfl][cal].axes = axes 
675              plot[cfl][cal].axes.set_title(cal) 
676      elif isinstance(axes, str): 
677          cal = axes 
678          if cal not in plot[cfl].keys(): 
679              plot[cfl][cal] = pargs() 
680              plot[cfl][cal].axes = pylab.axes() 
681              plot[cfl][cal].axes.set_title(cal) 
682   
683      pylab.axes(plot[cfl][cal].axes) 
684   
685      plot._cfl = cfl 
686      plot._cal = cal

687
```

  


| Home | Trees | Indices | Help | PyDSTool |
| --- | --- | --- | --- | --- |

|  |  |
| --- | --- |
| Generated by Epydoc 3.0.1 on Fri May 4 15:24:14 2012 | http://epydoc.sourceforge.net |
